# Supplementary material for: Genetic Associations of ITGB3, FGG, GP1BA, PECAM1, and PEAR1 Polymorphisms and the Platelet Activation Pathway with Recurrent Pregnancy Loss in the Korean Population
Source: Int J Mol Sci. 2025 Aug 3;26(15):7505. doi: 10.3390/ijms26157505 (PMC12347128; doi:10.3390/ijms26157505)
Supplement: Supplementary file 1 [file ijms-26-07505-s001.zip › ijms-3747198-supplementary.pdf]

Supplementary Table S1. The information of SNPs analyzed in this study.

| Gene | Full name | Chr | rs number | position | strand | Effect |
|---|---|---|---|---|---|---|
| *ITGB3* | integrin subunit beta 3 | Chr17 | rs2317676 | 47310917 | + | 3' UTR |
| | | | rs3809865 | 47311220 | + | 3' UTR |
| *FGG* | fibrinogen gamma chain | Chr4 | rs1049636 | 154604818 | - | 3' UTR |
| | | | rs2066865 | 154604124 | - | 500b downstream |
| *GP1BA* | glycoprotein Ib platelet subunit alpha | Chr17 | rs2243093 | 4932600 | + | 5' UTR |
| | | | rs6065 | 4933086 | + | missense<br>Thr(ACG)161Met(AGG) |
| *PECAM1* | platelet and endothelial cell adhesion molecule 1 | Chr17 | rs2812 | 64323758 | - | 3' UTR |
| *PEAR1* | platelet endothelial aggregation receptor 1 | Chr1 | rs822442 | 156913423 | + | missense<br>Asn(AAC)848Lys(AAA) |
| | | | rs12137505 | 156913754 | + | missense<br>Asn(AAT)903Asp(GAT) |

The position information was based on the GRCh38 reference genome.

Supplementary Table S2. Comparison of genotype frequencies of *ITGB3*, *FGG*, *GP1BA*, *PECAM1*, *PEAR1* polymorphisms between the RPL and control subjects.

| Genotypes | Controls (n=375) | RPL (n=389) | AOR (95% CI) | *P* | FDR-*P* | PL≥3 (n=205) | AOR (95% CI) | *P* | FDR-*P* | PL≥4 (n=76) | AOR (95% CI) | *P* | FDR-*P* |
|---|---|---|---|---|---|---|---|---|---|---|---|---|---|
| *ITGB3* rs2317676A>G | | | | | | | | | | | | | |
| AA | 255 (68.0) | 249 (64.0) | 1.000 (reference) | | | 106 (51.7) | 1.000 (reference) | | | 45 (59.2) | 1.000 (reference) | | |
| AG | 110 (29.3) | 122 (31.4) | 1.121 (0.820-1.532) | 0.474 | 0.853 | 87 (42.4) | 1.079 (0.742-1.570) | 0.690 | 0.755 | 27 (35.5) | 1.004 (0.584-1.726) | 0.990 | |
| GG | 10 (2.7) | 18 (4.6) | 1.942 (0.874-4.313) | 0.103 | 0.351 | 12 (5.9) | 1.318 (0.462-3.761) | 0.605 | 0.790 | 4 (5.3) | 0.538 (0.067-4.327) | 0.56 | 0.841 |
| Dominant (AA vs AG+GG) | | | 1.184 (0.876-1.600) | 0.271 | 0.61 | | 1.092 (0.759-1.570) | 0.637 | 0.712 | | 0.964 (0.566-1.641) | 0.892 | 0.892 |
| Recessive (AA+AG vs GG) | | | 1.824 (0.829-4.011) | 0.135 | 0.608 | | 1.206 (0.429-3.388) | 0.722 | 0.939 | | 0.509 (0.064-4.049) | 0.523 | 0.945 |
| HWE-*P* | 0.648 | 0.54 | | | | | | | | | | | |
| *FGG* rs2066865T>C | | | | | | | | | | | | | |
| TT | 95 (25.3) | 99 (25.4) | 1.000 (reference) | | | 48 (23.4) | 1.000 (reference) | | | 18 (23.7) | 1.000 (reference) | | |
| TC | 195 (52.0) | 195 (50.1) | 0.901 (0.632-1.285) | 0.566 | 0.896 | 108 (52.7) | 0.987 (0.644-1.511) | 0.950 | 0.755 | 35 (46.1) | 0.666 (0.371-1.196) | 0.174 | 0.99 |
| CC | 85 (22.7) | 95 (24.4) | 0.934 (0.621-1.404) | 0.741 | 0.926 | 49 (23.9) | 0.907 (0.551-1.493) | 0.702 | 0.790 | 23 (30.3) | 0.703 (0.355-1.393) | 0.312 | 0.841 |
| Dominant (TT vs TC+CC) | | | 0.915 (0.654-1.279) | 0.602 | 0.949 | | 0.965 (0.643-1.447) | 0.862 | 0.712 | | 0.683 (0.395-1.180) | 0.172 | 0.892 |
| Recessive (TT+TC vs CC) | | | 1.011 (0.729-1.403) | 0.949 | 0.797 | | 0.927 (0.621-1.384) | 0.712 | 0.939 | | 0.923 (0.518-1.647) | 0.787 | 0.774 |
| HWE-*P* | 0.43 | 0.958 | | | | | | | | | | | |
| *GP1BA* rs2243093T>C | | | | | | | | | | | | | |
| TT | 202 (53.9) | 214 (55.0) | 1.000 (reference) | | | 135 (65.9) | 1.000 (reference) | | | 52 (68.4) | 1.000 (reference) | | |
| TC | 149 (39.7) | 154 (39.6) | 0.980 (0.728-1.320) | 0.896 | 0.896 | 64 (31.2) | 1.124 (0.787-1.605) | 0.521 | 0.67 | 23 (30.3) | 0.822 (0.487-1.386) | 0.461 | 0.990 |
| CC | 24 (6.4) | 21 (5.4) | 0.836 (0.450-1.551) | 0.57 | 0.926 | 6 (2.9) | 0.986 (0.473-2.057) | 0.970 | 0.970 | 1 (1.3) | 0.749 (0.247-2.268) | 0.609 | 0.841 |
| Dominant (TT vs TC+CC) | | | 0.964 (0.724-1.282) | 0.799 | 0.949 | | 1.108 (0.786-1.561) | 0.559 | 0.712 | | 0.812 (0.492-1.341) | 0.417 | 0.892 |
| Recessive (TT+TC vs CC) | | | 0.865 (0.472-1.585) | 0.639 | 0.797 | | 0.960 (0.468-1.971) | 0.911 | 0.939 | | 0.825 (0.277-2.452) | 0.729 | 0.945 |
| HWE-*P* | 0.618 | 0.321 | | | | | | | | | | | |
| *GP1BA* rs6065C>T | | | | | | | | | | | | | |
| CC | 312 (83.2) | 315 (81.0) | 1.000 (reference) | | | 164 (80.0) | 1.000 (reference) | | | 62 (81.6) | 1.000 (reference) | | |
| CT | 58 (15.5) | 71 (18.3) | 1.209 (0.825-1.771) | 0.331 | 0.745 | 40 (19.5) | 1.317 (0.841-2.062) | 0.229 | 0.670 | 13 (17.1) | 1.133 (0.585-2.195) | 0.711 | 0.990 |
| TT | 5 (1.3) | 3 (0.8) | 0.511 (0.118-2.217) | 0.370 | 0.833 | 1 (0.5) | 0.242 (0.025-2.386) | 0.224 | 0.672 | 1 (1.3) | 0.790 (0.083-7.533) | 0.837 | 0.845 |
| Dominant (CC vs CT+TT) | | | 1.152 (0.794-1.670) | 0.457 | 0.823 | | 1.220 (0.786-1.893) | 0.376 | 0.712 | | 1.104 (0.581-2.097) | 0.763 | 0.892 |
| Recessive (CC+CT vs TT) | | | 0.487 (0.112-2.114) | 0.337 | 0.758 | | 0.234 (0.024-2.274) | 0.210 | 0.747 | | 0.804 (0.086-7.504) | 0.848 | 0.945 |
| HWE-*P* | 0.23 | 0.645 | | | | | | | | | | | |
| *PECAM1* rs2812 C>T | | | | | | | | | | | | | |
| CC | 168 (44.8) | 176 (45.2) | 1.000 (reference) | | | 98 (47.8) | 1.000 (reference) | | | 39 (51.3) | 1.000 (reference) | | |
| CT | 164 (43.7) | 168 (43.2) | 0.978 (0.723-1.322) | 0.883 | 0.896 | 84 (41.0) | 0.873 (0.607-1.255) | 0.463 | 0.670 | 29 (38.2) | 0.760 (0.449-1.287) | 0.308 | 0.990 |
| TT | 43 (11.5) | 45 (11.6) | 0.972 (0.607-1.559) | 0.907 | 0.926 | 23 (11.2) | 0.891 (0.505-1.574) | 0.692 | 0.790 | 8 (10.5) | 0.800 (0.348-1.838) | 0.598 | 0.841 |
| Dominant (CC vs CT+TT) | | | 0.977 (0.734-1.300) | 0.870 | 0.949 | | 0.875 (0.621-1.234) | 0.447 | 0.712 | | 0.768 (0.468-1.258) | 0.294 | 0.882 |
| Recessive (CC+CT vs TT) | | | 0.984 (0.629-1.540) | 0.945 | 0.945 | 280(68.3) | 0.953 (0.555-1.638) | 0.862 | 0.939 | | 0.908 (0.408-2.019) | 0.813 | 0.945 |
| HWE-*P* | 0.757 | 0.611 | | | | 86 (42.0) | | | | | | | |
| *PEAR1* rs822442C>A | | | | | | 93 (45.4) | | | | | | | |
| CC | 185 (49.3) | 169 (43.4) | 1.000 (reference) | | | 26 (12.7) | 1.000 (reference) | | | 39 (51.3) | 1.000 (reference) | | |
| CA | 154 (41.1) | 172 (44.2) | 1.228 (0.907-1.662) | 0.184 | 0.745 | | 1.281 (0.888-1.847) | 0.185 | 0.670 | 28 (36.8) | 0.865 (0.509-1.470) | 0.591 | 0.990 |
| AA | 36 (9.6) | 48 (12.3) | 1.468 (0.909-2.373) | 0.117 | 0.351 | | 1.554 (0.883-2.737) | 0.126 | 0.567 | 9 (11.8) | 1.204 (0.535-2.706) | 0.654 | 0.841 |
| Dominant (CC vs CA+AA) | | | 1.275 (0.958-1.697) | 0.096 | 0.288 | 265 (64.6) | 1.334 (0.945 - 1.884) | 0.102 | 0.306 | | 0.921 (0.562 - 1.509) | 0.744 | 0.892 |
| Recessive (CC+CA vs AA) | | | 1.340 (0.847-2.120) | 0.211 | 0.633 | | 1.374 (0.800-2.359) | 0.249 | 0.747 | | 1.230 (0.563-2.684) | 0.604 | 0.945 |
| HWE-*P* | 0.633 | 0.679 | | | | 38 (18.5) | | | | | | | |
| *PEAR1* rs12137505G>A | | | | | | | | | | | | | |
| GG | 118 (31.5) | 118 (30.3) | 1.000 (reference) | | | | 1.000 (reference) | | | 25 (32.9) | 1.000 (reference) | | |
| GA | 188 (50.1) | 204 (52.4) | 1.092 (0.790-1.511) | 0.594 | 0.891 | 226 (55.1) | 1.152 (0.776-1.709) | 0.483 | 0.670 | 39 (51.3) | 0.981 (0.565-1.706) | 0.947 | 0.990 |
| AA | 69 (18.4) | 67 (17.2) | 0.980 (0.643-1.496) | 0.926 | 0.926 | 184 (44.9) | 1.105 (0.667-1.831) | 0.698 | 0.790 | 12 (15.8) | 0.821 (0.388-1.737) | 0.606 | 0.841 |
| Dominant (GG vs GA+AA) | | | 1.064 (0.782-1.447) | 0.695 | 0.949 | | 1.141 (0.784-1.660) | 0.491 | 0.712 | | 0.938 (0.554-1.588) | 0.812 | 0.892 |
| Recessive (GG+GA vs AA) | | | 0.931 (0.642-1.351) | 0.708 | 0.797 | | 1.017 (0.654-1.582) | 0.939 | 0.939 | | 0.825 (0.422-1.613) | 0.573 | 0.945 |
| HWE-*P* | 0.697 | 0.185 | | | | | | | | | | | |

RPL, recurrent pregnancy loss; COR, crude odds ratio; AOR, adjusted odds ratio; FDR, false discovery rate; HWE, Hardy-Weinberg equilibrium; adjusted by age.

Supplementary Table S3. Allele combination analysis of *ITGB3*, *FGG*, *GP1BA*, *PECAM1*, *PEAR1* gene polymorphisms in RPL and controls subjects.

| Allele combination | Controls (2n=750) | RPL (2n=778) | OR (95% CI) | *P* | FDR-*P* |
|---|---|---|---|---|---|
| *ITGB3* rs2317676A>G/*ITGB3* rs3809865A>T/*FGG* rs1049636T>C/*FGG* rs2066865T>C/*GP1BA* rs2243093T>C/ *GP1BA* rs6065C>T/*PECAM1* rs2812C>T/*PEAR1* rs822442C>A/*PEAR1* rs12137505G>A | | | | | |
| A.A.T.T.T.C.C.C.G | 43 (5.7) | 55 (7.1) | 1.000 (reference) | | |
| A.A.T.T.T.C.C.C.A | 14 (1.9) | 5 (0.6) | 0.279 (0.093-0.836) | 0.017 | 0.149 |
| A.A.T.T.T.C.C.A.G | 0 (0.0) | 1 (0.2) | 2.351 (0.093-59.200) | 1.000 | 1.000 |
| A.A.T.T.T.C.C.A.A | 50 (6.7) | 35 (4.5) | 0.547 (0.304-0.986) | 0.044 | 0.221 |
| A.A.T.T.T.C.T.C.G | 44 (5.9) | 44 (5.7) | 0.782 (0.439-1.393) | 0.403 | 0.759 |
| A.A.T.T.T.C.T.A.A | 17 (2.3) | 0 (0.0) | 0.022 (0.001-0.383) | 0.0001 | 0.008 |
| A.A.T.T.T.T.C.C.G | 5 (0.7) | 11 (1.4) | 1.720 (0.556-5.326) | 0.343 | 0.714 |
| A.A.T.T.T.T.C.C.A | 7 (0.9) | 0 (0.0) | 0.052 (0.003-0.941) | 0.004 | 0.051 |
| A.A.T.T.T.T.C.A.A | 0 (0.0) | 7 (1.0) | 11.760 (0.653-211.700) | 0.040 | 0.220 |
| A.A.T.T.T.T.T.C.G | 2 (0.3) | 0 (0.0) | 0.157 (0.007-3.353) | 0.200 | 0.498 |
| A.A.T.T.T.T.T.A.A | 0 (0.0) | 2 (0.2) | 3.919 (0.183-83.830) | 0.505 | 0.763 |
| A.A.T.T.C.C.C.C.G | 21 (2.8) | 22 (2.8) | 0.819 (0.399-1.681) | 0.586 | 0.840 |
| A.A.T.T.C.C.C.C.A | 3 (0.4) | 4 (0.6) | 1.042 (0.221-4.909) | 1.000 | 1.000 |
| A.A.T.T.C.C.C.A.A | 5 (0.6) | 16 (2.0) | 2.502 (0.849-7.373) | 0.089 | 0.313 |
| A.A.T.T.C.C.T.C.G | 8 (1.0) | 10 (1.3) | 0.977 (0.355-2.688) | 0.965 | 1.000 |
| A.A.T.T.C.C.T.C.A | 5 (0.7) | 0 (0.0) | 0.071 (0.004-1.325) | 0.020 | 0.149 |
| A.A.T.T.C.C.T.A.A | 3 (0.4) | 2 (0.3) | 0.521 (0.083-3.261) | 0.654 | 0.904 |
| A.A.T.T.C.T.C.C.G | 0 (0.0) | 4 (0.5) | 7.054 (0.370-134.700) | 0.136 | 0.398 |
| A.A.T.T.C.T.C.C.A | 1 (0.2) | 0 (0.0) | 0.261 (0.010-6.578) | 0.444 | 0.759 |
| A.A.T.C.T.C.C.C.G | 54 (7.2) | 32 (4.1) | 0.463 (0.256-0.838) | 0.010 | 0.099 |
| A.A.T.C.T.C.C.C.A | 24 (3.1) | 8 (1.0) | 0.261 (0.107-0.637) | 0.002 | 0.051 |
| A.A.T.C.T.C.C.A.G | 1 (0.2) | 0 (0.0) | 0.261 (0.010-6.578) | 0.444 | 0.759 |
| A.A.T.C.T.C.C.A.A | 9 (1.3) | 28 (3.6) | 2.432 (1.039-5.694) | 0.037 | 0.220 |
| A.A.T.C.T.C.T.C.G | 7 (0.9) | 10 (1.3) | 1.117 (0.393-3.177) | 0.836 | 1.000 |
| A.A.T.C.T.C.T.C.A | 2 (0.2) | 16 (2.1) | 6.255 (1.363-28.700) | 0.009 | 0.093 |
| A.A.T.C.T.C.T.A.A | 9 (1.1) | 14 (1.8) | 1.216 (0.481-3.076) | 0.679 | 0.907 |
| A.A.T.C.T.T.C.C.G | 3 (0.4) | 4 (0.6) | 1.042 (0.221-4.909) | 1.000 | 1.000 |
| A.A.T.C.T.T.C.A.A | 2 (0.2) | 3 (0.4) | 1.173 (0.187-7.337) | 1.000 | 1.000 |
| A.A.T.C.T.T.T.C.G | 1 (0.2) | 4 (0.5) | 3.127 (0.337-29.020) | 0.390 | 0.759 |
| A.A.T.C.C.C.C.C.G | 4 (0.5) | 5 (0.7) | 0.977 (0.247-3.863) | 1.000 | 1.000 |
| A.A.T.C.C.C.C.C.A | 2 (0.3) | 4 (0.6) | 1.564 (0.273-8.945) | 0.696 | 0.907 |
| A.A.T.C.C.C.C.A.G | 0 (0.0) | 2 (0.2) | 3.919 (0.183-83.830) | 0.505 | 0.763 |
| A.A.T.C.C.C.C.A.A | 30 (4.0) | 4 (0.6) | 0.104 (0.034-0.319) | 0.0001 | 0.008 |
| A.A.T.C.C.C.T.C.G | 11 (1.4) | 10 (1.3) | 0.711 (0.276-1.829) | 0.478 | 0.763 |
| A.A.C.T.T.C.C.C.G | 1 (0.2) | 0 (0.0) | 0.261 (0.010-6.578) | 0.444 | 0.759 |
| A.A.C.T.T.T.T.A.G | 0 (0.0) | 1 (0.1) | 2.351 (0.093-59.200) | 1.000 | 1.000 |
| A.A.C.T.C.C.T.C.G | 0 (0.0) | 3 (0.4) | 5.486 (0.276-109.100) | 0.259 | 0.579 |
| A.A.C.C.T.C.C.C.G | 24 (3.2) | 23 (3.0) | 0.749 (0.373-1.505) | 0.417 | 0.759 |
| A.A.C.C.T.C.C.C.A | 6 (0.8) | 4 (0.6) | 0.521 (0.138-1.965) | 0.507 | 0.763 |
| A.A.C.C.T.C.C.A.G | 0 (0.0) | 4 (0.6) | 7.054 (0.370-134.700) | 0.136 | 0.398 |
| A.A.C.C.T.C.C.A.A | 12 (1.6) | 7 (0.9) | 0.456 (0.165-1.257) | 0.123 | 0.398 |
| A.A.C.C.T.C.T.C.G | 5 (0.7) | 4 (0.5) | 0.626 (0.158-2.472) | 0.728 | 0.931 |
| A.A.C.C.T.C.T.C.A | 3 (0.4) | 0 (0.0) | 0.112 (0.006-2.227) | 0.091 | 0.313 |
| A.A.C.C.T.C.T.A.A | 7 (0.9) | 12 (1.5) | 1.340 (0.486-3.695) | 0.571 | 0.840 |
| A.A.C.C.T.T.C.C.A | 0 (0.0) | 1 (0.2) | 2.351 (0.093-59.200) | 1.000 | 1.000 |
| A.A.C.C.T.T.C.A.A | 2 (0.2) | 5 (0.7) | 1.955 (0.361-10.570) | 0.696 | 0.907 |
| A.A.C.C.T.T.T.A.A | 3 (0.4) | 0 (0.0) | 0.112 (0.006-2.227) | 0.091 | 0.313 |
| A.A.C.C.C.C.C.C.G | 1 (0.2) | 0 (0.0) | 0.261 (0.010-6.578) | 0.444 | 0.759 |
| A.A.C.C.C.C.C.C.A | 7 (0.9) | 0 (0.0) | 0.052 (0.003-0.941) | 0.004 | 0.051 |
| A.A.C.C.C.C.C.A.G | 0 (0.0) | 2 (0.3) | 3.919 (0.183-83.830) | 0.505 | 0.763 |
| A.A.C.C.C.C.C.A.A | 4 (0.6) | 4 (0.6) | 0.782 (0.185-3.308) | 1.000 | 1.000 |
| A.A.C.C.C.C.T.C.G | 8 (1.1) | 3 (0.4) | 0.293 (0.073-1.172) | 0.069 | 0.313 |
| A.A.C.C.C.C.T.A.G | 0 (0.0) | 1 (0.1) | 2.351 (0.093-59.200) | 1.000 | 1.000 |
| A.A.C.C.C.C.T.A.A | 3 (0.4) | 0 (0.0) | 0.112 (0.006-2.227) | 0.091 | 0.313 |
| A.A.C.C.C.T.C.C.G | 2 (0.3) | 0 (0.0) | 0.157 (0.007-3.353) | 0.200 | 0.498 |
| A.A.C.C.C.T.T.C.A | 0 (0.0) | 1 (0.1) | 2.351 (0.093-59.200) | 1.000 | 1.000 |
| A.T.T.T.T.C.C.C.G | 36 (4.8) | 11 (1.4) | 0.239 (0.109-0.524) | 0.0002 | 0.010 |
| A.T.T.T.T.C.C.C.A | 0 (0.0) | 4 (0.5) | 7.054 (0.370-134.700) | 0.136 | 0.398 |
| A.T.T.T.T.C.C.A.G | 1 (0.2) | 3 (0.4) | 2.345 (0.236-23.360) | 0.632 | 0.889 |
| A.T.T.T.T.C.C.A.A | 14 (1.8) | 20 (2.5) | 1.117 (0.506-2.464) | 0.784 | 0.993 |
| A.T.T.T.T.C.T.C.G | 3 (0.4) | 10 (1.3) | 2.606 (0.675-10.060) | 0.153 | 0.430 |
| A.T.T.T.T.C.T.C.A | 0 (0.0) | 3 (0.3) | 5.486 (0.276-109.100) | 0.259 | 0.579 |
| A.T.T.T.T.C.T.A.A | 6 (0.8) | 15 (1.9) | 1.955 (0.699-5.462) | 0.196 | 0.498 |
| A.T.T.T.T.T.C.C.G | 3 (0.4) | 6 (0.8) | 1.564 (0.370-6.616) | 0.729 | 0.931 |
| A.T.T.T.T.T.C.A.G | 0 (0.0) | 1 (0.1) | 2.351 (0.093-59.200) | 1.000 | 1.000 |
| A.T.T.T.T.T.T.C.G | 4 (0.5) | 0 (0.0) | 0.087 (0.005-1.663) | 0.042 | 0.220 |
| A.T.T.T.T.T.T.C.A | 5 (0.6) | 0 (0.0) | 0.071 (0.004-1.325) | 0.020 | 0.149 |
| A.T.T.T.C.C.C.C.G | 0 (0.0) | 11 (1.5) | 18.030 (1.033-314.700) | 0.003 | 0.051 |
| A.T.T.T.C.C.C.C.A | 3 (0.5) | 1 (0.2) | 0.261 (0.026-2.596) | 0.325 | 0.686 |

| | | | | | |
|---|---|---|---|---|---|
| A.T.T.T.C.C.C.A.A | 7 (0.9) | 0 (0.0) | 0.052 (0.003-0.941) | 0.004 | 0.051 |
| A.T.T.T.C.C.T.C.A | 3 (0.4) | 0 (0.0) | 0.112 (0.006-2.227) | 0.091 | 0.313 |
| A.T.T.T.C.T.C.A.A | 2 (0.2) | 0 (0.0) | 0.157 (0.007-3.353) | 0.200 | 0.498 |
| A.T.T.T.C.T.T.C.G | 0 (0.0) | 1 (0.2) | 2.351 (0.093-59.200) | 1.000 | 1.000 |
| A.T.T.C.T.C.C.C.G | 0 (0.0) | 17 (2.2) | 27.430 (1.603-469.400) | 0.001 | 0.023 |
| A.T.T.C.T.C.C.C.A | 3 (0.4) | 7 (0.9) | 1.824 (0.445-7.476) | 0.512 | 0.763 |
| A.T.T.C.T.C.C.A.G | 0 (0.0) | 1 (0.2) | 2.351 (0.093-59.200) | 1.000 | 1.000 |
| A.T.T.C.T.C.C.A.A | 4 (0.5) | 6 (0.8) | 1.173 (0.311-4.420) | 1.000 | 1.000 |
| A.T.T.C.T.C.T.C.G | 6 (0.8) | 12 (1.5) | 1.564 (0.543-4.506) | 0.405 | 0.759 |
| A.T.T.C.T.T.T.C.G | 5 (0.6) | 1 (0.2) | 0.156 (0.018-1.389) | 0.093 | 0.313 |
| A.T.T.C.C.C.C.C.G | 0 (0.0) | 2 (0.3) | 3.919 (0.183-83.830) | 0.505 | 0.763 |
| A.T.T.C.C.C.C.A.A | 2 (0.3) | 3 (0.4) | 1.173 (0.187-7.337) | 1.000 | 1.000 |
| A.T.T.C.C.C.T.C.G | 0 (0.0) | 5 (0.7) | 8.622 (0.464-160.300) | 0.073 | 0.313 |
| A.T.T.C.C.C.T.A.A | 0 (0.0) | 12 (1.6) | 19.590 (1.128-340.500) | 0.003 | 0.051 |
| A.T.T.C.C.T.T.C.A | 0 (0.0) | 1 (0.1) | 2.351 (0.093-59.200) | 1.000 | 1.000 |
| A.T.C.T.C.C.C.A.G | 1 (0.1) | 0 (0.0) | 0.261 (0.010-6.578) | 0.444 | 0.759 |
| A.T.C.T.C.C.T.A.A | 1 (0.1) | 1 (0.2) | 0.782 (0.047-12.870) | 1.000 | 1.000 |
| A.T.C.C.T.C.C.C.G | 7 (0.9) | 11 (1.4) | 1.229 (0.439-3.436) | 0.694 | 0.907 |
| A.T.C.C.T.C.C.A.A | 1 (0.2) | 0 (0.0) | 0.261 (0.010-6.578) | 0.444 | 0.759 |
| A.T.C.C.T.C.T.C.G | 8 (1.0) | 3 (0.4) | 0.293 (0.073-1.172) | 0.069 | 0.313 |
| A.T.C.C.T.C.T.C.A | 1 (0.1) | 0 (0.0) | 0.261 (0.010-6.578) | 0.444 | 0.759 |
| A.T.C.C.T.C.T.A.A | 1 (0.1) | 3 (0.4) | 2.345 (0.236-23.360) | 0.632 | 0.889 |
| A.T.C.C.T.T.C.C.G | 0 (0.0) | 3 (0.4) | 5.486 (0.276-109.100) | 0.259 | 0.579 |
| A.T.C.C.T.T.T.C.G | 5 (0.6) | 0 (0.0) | 0.071 (0.004-1.325) | 0.020 | 0.149 |
| A.T.C.C.C.C.C.C.G | 9 (1.1) | 0 (0.0) | 0.041 (0.002-0.729) | 0.001 | 0.030 |
| A.T.C.C.C.C.T.C.G | 6 (0.8) | 0 (0.0) | 0.060 (0.003-1.101) | 0.009 | 0.093 |
| A.T.C.C.C.C.T.C.A | 2 (0.3) | 1 (0.2) | 0.391 (0.034-4.458) | 0.584 | 0.840 |
| A.T.C.C.C.T.C.C.A | 1 (0.1) | 0 (0.0) | 0.261 (0.010-6.578) | 0.444 | 0.759 |
| A.T.C.C.C.T.C.A.A | 1 (0.1) | 2 (0.3) | 1.564 (0.137-17.830) | 1.000 | 1.000 |
| G.A.T.T.T.C.C.C.G | 19 (2.5) | 21 (2.7) | 0.864 (0.413-1.807) | 0.698 | 0.907 |
| G.A.T.T.T.C.C.C.A | 0 (0.0) | 4 (0.5) | 7.054 (0.370-134.700) | 0.136 | 0.398 |
| G.A.T.T.T.C.C.A.A | 3 (0.4) | 0 (0.0) | 0.112 (0.006-2.227) | 0.091 | 0.313 |
| G.A.T.T.T.C.T.C.G | 7 (1.0) | 0 (0.0) | 0.052 (0.003-0.941) | 0.004 | 0.051 |
| G.A.T.T.T.C.T.C.A | 6 (0.7) | 2 (0.2) | 0.261 (0.050-1.357) | 0.140 | 0.402 |
| G.A.T.T.T.C.T.A.G | 0 (0.0) | 1 (0.2) | 2.351 (0.093-59.200) | 1.000 | 1.000 |
| G.A.T.T.T.C.T.A.A | 7 (0.9) | 7 (0.9) | 0.782 (0.255-2.399) | 0.666 | 0.907 |
| G.A.T.T.T.T.C.C.G | 2 (0.3) | 0 (0.0) | 0.157 (0.007-3.353) | 0.200 | 0.498 |
| G.A.T.T.T.T.C.C.A | 3 (0.4) | 2 (0.3) | 0.521 (0.083-3.261) | 0.654 | 0.904 |
| G.A.T.T.T.T.T.A.A | 0 (0.0) | 2 (0.2) | 3.919 (0.183-83.830) | 0.505 | 0.763 |
| G.A.T.T.C.C.C.C.G | 14 (1.9) | 18 (2.4) | 1.005 (0.450-2.247) | 0.990 | 1.000 |
| G.A.T.T.C.C.C.C.A | 0 (0.0) | 7 (1.0) | 11.760 (0.653-211.700) | 0.040 | 0.220 |
| G.A.T.T.C.C.C.A.G | 0 (0.0) | 1 (0.2) | 2.351 (0.093-59.200) | 1.000 | 1.000 |
| G.A.T.T.C.C.C.A.A | 0 (0.0) | 4 (0.6) | 7.054 (0.370-134.700) | 0.136 | 0.398 |
| G.A.T.T.C.C.T.C.A | 3 (0.4) | 1 (0.2) | 0.261 (0.026-2.596) | 0.325 | 0.686 |
| G.A.T.T.C.C.T.A.A | 0 (0.0) | 2 (0.3) | 3.919 (0.183-83.830) | 0.505 | 0.763 |
| G.A.T.T.C.T.T.A.A | 0 (0.0) | 1 (0.1) | 2.351 (0.093-59.200) | 1.000 | 1.000 |
| G.A.T.C.T.C.C.C.G | 4 (0.5) | 3 (0.4) | 0.586 (0.125-2.761) | 0.698 | 0.907 |
| G.A.T.C.T.C.C.C.A | 0 (0.0) | 2 (0.3) | 3.919 (0.183-83.830) | 0.505 | 0.763 |
| G.A.T.C.T.C.C.A.A | 3 (0.4) | 1 (0.1) | 0.261 (0.026-2.596) | 0.325 | 0.686 |
| G.A.T.C.T.C.T.C.G | 13 (1.7) | 5 (0.7) | 0.301 (0.099-0.909) | 0.027 | 0.195 |
| G.A.T.C.T.C.T.A.A | 0 (0.0) | 5 (0.6) | 8.622 (0.464-160.300) | 0.073 | 0.313 |
| G.A.T.C.T.T.C.C.G | 0 (0.0) | 7 (0.9) | 11.760 (0.653-211.700) | 0.040 | 0.220 |
| G.A.T.C.T.T.C.A.A | 1 (0.2) | 0 (0.0) | 0.261 (0.010-6.578) | 0.444 | 0.759 |
| G.A.T.C.T.T.T.A.A | 2 (0.3) | 0 (0.0) | 0.157 (0.007-3.353) | 0.200 | 0.498 |
| G.A.T.C.C.C.C.C.G | 11 (1.5) | 4 (0.6) | 0.284 (0.085-0.956) | 0.033 | 0.220 |
| G.A.T.C.C.C.C.C.A | 0 (0.0) | 4 (0.5) | 7.054 (0.370-134.700) | 0.136 | 0.398 |
| G.A.T.C.C.C.T.A.A | 2 (0.2) | 3 (0.4) | 1.173 (0.187-7.337) | 1.000 | 1.000 |
| G.A.C.T.T.C.C.A.G | 1 (0.1) | 1 (0.1) | 0.782 (0.047-12.870) | 1.000 | 1.000 |
| G.A.C.T.C.C.C.C.G | 0 (0.0) | 1 (0.2) | 2.351 (0.093-59.200) | 1.000 | 1.000 |
| G.A.C.C.T.C.C.C.G | 2 (0.3) | 0 (0.0) | 0.157 (0.007-3.353) | 0.200 | 0.498 |
| G.A.C.C.T.C.C.A.A | 0 (0.0) | 11 (1.4) | 18.030 (1.033-314.700) | 0.003 | 0.051 |
| G.A.C.C.T.C.T.C.G | 2 (0.3) | 7 (0.9) | 2.736 (0.541-13.850) | 0.298 | 0.656 |
| G.A.C.C.T.C.T.A.G | 0 (0.0) | 2 (0.3) | 3.919 (0.183-83.830) | 0.505 | 0.763 |
| G.A.C.C.T.C.T.A.A | 3 (0.4) | 0 (0.0) | 0.112 (0.006-2.227) | 0.091 | 0.313 |
| G.A.C.C.T.T.C.C.G | 0 (0.0) | 3 (0.4) | 5.486 (0.276-109.100) | 0.259 | 0.579 |
| G.A.C.C.T.T.C.C.A | 0 (0.0) | 1 (0.2) | 2.351 (0.093-59.200) | 1.000 | 1.000 |
| G.A.C.C.T.T.T.C.G | 4 (0.6) | 0 (0.0) | 0.087 (0.005-1.663) | 0.042 | 0.220 |
| G.A.C.C.C.C.C.A.A | 3 (0.4) | 0 (0.0) | 0.112 (0.006-2.227) | 0.091 | 0.313 |
| G.A.C.C.C.C.T.C.G | 3 (0.4) | 0 (0.0) | 0.112 (0.006-2.227) | 0.091 | 0.313 |
| G.A.C.C.C.C.T.C.A | 0 (0.0) | 2 (0.3) | 3.919 (0.183-83.830) | 0.505 | 0.763 |
| G.A.C.C.C.C.T.A.G | 0 (0.0) | 1 (0.1) | 2.351 (0.093-59.200) | 1.000 | 1.000 |
| G.A.C.C.C.C.T.A.A | 4 (0.5) | 0 (0.0) | 0.087 (0.005-1.663) | 0.042 | 0.220 |
| G.T.T.T.T.C.C.C.G | 0 (0.0) | 3 (0.4) | 5.486 (0.276-109.100) | 0.259 | 0.579 |
| G.T.T.T.T.C.T.C.G | 0 (0.0) | 3 (0.4) | 5.486 (0.276-109.100) | 0.259 | 0.579 |

| | | | | | |
|---|---|---|---|---|---|
| G.T.T.T.T.T.T.A.A | 2 (0.3) | 1 (0.1) | 0.391 (0.034-4.458) | 0.584 | 0.840 |
| G.T.T.T.C.C.T.C.G | 1 (0.2) | 1 (0.2) | 0.782 (0.047-12.870) | 1.000 | 1.000 |
| G.T.T.C.T.C.C.A.A | 0 (0.0) | 2 (0.3) | 3.919 (0.183-83.830) | 0.505 | 0.763 |
| G.T.T.C.C.C.T.C.A | 1 (0.1) | 0 (0.0) | 0.261 (0.010-6.578) | 0.444 | 0.759 |
| G.T.T.C.C.C.T.A.G | 0 (0.0) | 1 (0.1) | 2.351 (0.093-59.200) | 1.000 | 1.000 |
| G.T.C.C.T.C.C.C.A | 0 (0.0) | 5 (0.7) | 8.622 (0.464-160.300) | 0.073 | 0.313 |
| G.T.C.C.T.C.C.A.A | 1 (0.2) | 0 (0.0) | 0.261 (0.010-6.578) | 0.444 | 0.759 |
| G.T.C.C.T.C.T.A.A | 0 (0.0) | 3 (0.4) | 5.486 (0.276-109.100) | 0.259 | 0.579 |
| G.T.C.C.C.C.T.A.A | 0 (0.0) | 1 (0.1) | 2.351 (0.093-59.200) | 1.000 | 1.000 |
| G.T.C.C.C.T.C.A.A | 1 (0.2) | 0 (0.0) | 0.261 (0.010-6.578) | 0.444 | 0.759 |
| *ITGB3* rs2317676A>G/*ITGB3* rs3809865A>T/*FGG* rs1049636T>C/*PEAR1* rs12137505G>A | | | | | |
| A.A.T.G | 196 (26.2) | 209 (26.8) | 1.000 (reference) | | |
| A.A.T.A | 174 (23.3) | 141 (18.1) | 0.760 (0.566-1.021) | 0.068 | 0.319 |
| A.A.C.G | 48 (6.4) | 47 (6.1) | 0.918 (0.587-1.436) | 0.708 | 0.708 |
| A.A.C.A | 46 (6.2) | 38 (4.9) | 0.775 (0.483-1.242) | 0.288 | 0.502 |
| A.T.T.G | 65 (8.7) | 87 (11.1) | 1.255 (0.862-1.828) | 0.235 | 0.471 |
| A.T.T.A | 56 (7.4) | 80 (10.2) | 1.340 (0.904-1.985) | 0.144 | 0.336 |
| A.T.C.G | 29 (3.9) | 12 (1.5) | 0.388 (0.193-0.782) | 0.006 | 0.045 |
| A.T.C.A | 4 (0.6) | 7 (0.9) | 1.641 (0.473-5.695) | 0.431 | 0.538 |
| G.A.T.G | 72 (9.6) | 64 (8.3) | 0.834 (0.565-1.230) | 0.359 | 0.502 |
| G.A.T.A | 32 (4.2) | 51 (6.6) | 1.495 (0.922-2.423) | 0.102 | 0.336 |
| G.A.C.G | 13 (1.8) | 18 (2.3) | 1.298 (0.620-2.721) | 0.488 | 0.538 |
| G.A.C.A | 14 (1.8) | 10 (1.3) | 0.670 (0.291-1.544) | 0.344 | 0.502 |
| G.T.T.G | 0 (0.0) | 4 (0.5) | 8.442 (0.451-157.900) | 0.125 | 0.336 |
| G.T.T.A | 0 (0.0) | 2 (0.3) | 4.690 (0.224-98.370) | 0.499 | 0.538 |
| G.T.C.A | 0 (0.0) | 9 (1.2) | 17.820 (1.030-308.400) | 0.004 | 0.045 |
| *ITGB3* rs2317676A>G/*ITGB3* rs3809865A>T/*GP1BA* rs6065C>T | | | | | |
| A.A.C | 433 (57.7) | 382 (49.1) | 1.000 (reference) | | |
| A.A.T | 32 (4.3) | 51 (6.5) | 1.807 (1.137-2.870) | 0.011 | 0.023 |
| A.T.C | 129 (17.2) | 169 (21.8) | 1.485 (1.137-1.940) | 0.004 | 0.011 |
| A.T.T | 26 (3.4) | 19 (2.4) | 0.743 (0.410-1.346) | 0.325 | 0.390 |
| G.A.C | 120 (16.0) | 138 (17.7) | 1.304 (0.985-1.726) | 0.064 | 0.096 |
| G.A.T | 10 (1.4) | 8 (1.0) | 0.907 (0.354-2.322) | 0.838 | 0.838 |
| G.T.C | 0 (0.0) | 12 (1.6) | 28.330 (1.671-480.500) | 0.0003 | 0.002 |
| *ITGB3* rs3809865A>T/*GP1BA* rs6065C>T | | | | | |
| A.C | 553 (73.71) | 518 (66.64) | 1.000 (reference) | | |
| A.T | 42 (5.62) | 60 (7.65) | 1.525 (1.010 - 2.303) | 0.044 | 0.066 |
| T.C | 129 (17.22) | 183 (23.46) | 1.514 (1.173 - 1.955) | 0.001 | 0.003 |
| T.T | 26 (3.44) | 17 (2.24) | 0.698 (0.374 - 1.302) | 0.256 | 0.256 |

RPL, recurrent pregnancy loss; OR, odds ratio; CI, confidence interval

Supplementary Table S4. Genotype combination of *ITGB3, FGG, GP1BA, PECAM1, PEAR1* gene polymorphisms in RPL and controls subjects.

| Genotype combination | Controls (n=375) | RPL patients (n=389) | AOR (95% CI) | *P* | FDR-*P* |
|---|---|---|---|---|---|
| *ITGB3* rs3809865A>T/*FGG* rs1049636T>C/*PECAM1* rs2812C>T/*PEAR1* rs12137505G>A | | | | | |
| AA/TT/CC/GG | 19 (5.1) | 28 (7.2) | 1.000 (Reference) | | |
| AA/TT/CC/GA | 42 (11.2) | 35 (9.0) | 0.587 (0.280-1.232) | 0.159 | 0.753 |
| AA/TT/CC/AA | 17 (4.5) | 10 (2.6) | 0.403 (0.152-1.071) | 0.069 | 0.709 |
| AA/TT/CT/GG | 22 (5.9) | 11 (2.8) | 0.350 (0.137-0.893) | 0.028 | 0.581 |
| AA/TT/CT/GA | 24 (6.4) | 36 (9.3) | 1.095 (0.494-2.425) | 0.824 | 0.909 |
| AA/TT/CT/AA | 12 (3.2) | 9 (2.3) | 0.517 (0.179-1.492) | 0.223 | 0.753 |
| AA/TT/TT/GG | 6 (1.6) | 4 (1.0) | 0.452 (0.112-1.822) | 0.264 | 0.753 |
| AA/TT/TT/GA | 4 (1.1) | 11 (2.8) | 1.830 (0.505-6.637) | 0.358 | 0.753 |
| AA/TT/TT/AA | 3 (0.8) | 1 (0.3) | 0.240 (0.023-2.552) | 0.237 | 0.753 |
| AA/TC/CC/GG | 7 (1.9) | 7 (1.8) | 0.821 (0.235-2.862) | 0.757 | 0.909 |
| AA/TC/CC/GA | 18 (4.8) | 16 (4.1) | 0.617 (0.252-1.512) | 0.291 | 0.753 |
| AA/TC/CC/AA | 4 (1.1) | 3 (0.8) | 0.542 (0.107-2.752) | 0.460 | 0.784 |
| AA/TC/CT/GG | 11 (2.9) | 10 (2.6) | 0.637 (0.223-1.817) | 0.399 | 0.753 |
| AA/TC/CT/GA | 17 (4.5) | 13 (3.3) | 0.525 (0.206-1.338) | 0.177 | 0.753 |
| AA/TC/CT/AA | 7 (1.9) | 7 (1.8) | 0.671 (0.202-2.230) | 0.514 | 0.835 |
| AA/TC/TT/GG | 2 (0.5) | 4 (1.0) | 1.414 (0.233-8.573) | 0.706 | 0.909 |
| AA/TC/TT/GA | 5 (1.3) | 2 (0.5) | 0.291 (0.050-1.703) | 0.171 | 0.753 |
| AA/TC/TT/AA | 4 (1.1) | 0 (0.0) | N/A | N/A | N/A |
| AA/CC/CC/GG | 2 (0.5) | 1 (0.3) | 0.333 (0.028-3.961) | 0.384 | 0.753 |
| AA/CC/CC/GA | 1 (0.3) | 3 (0.8) | 1.999 (0.191-20.891) | 0.563 | 0.835 |
| AA/CC/CC/AA | 1 (0.3) | 0 (0.0) | N/A | N/A | N/A |
| AA/CC/CT/GG | 0 (0.0) | 4 (1.0) | N/A | N/A | N/A |
| AA/CC/CT/GA | 1 (0.3) | 1 (0.3) | 0.722 (0.042-12.443) | 0.823 | 0.909 |
| AA/CC/CT/AA | 2 (0.5) | 1 (0.3) | 0.367 (0.030-4.465) | 0.432 | 0.763 |
| AA/CC/TT/GG | 1 (0.3) | 0 (0.0) | N/A | N/A | N/A |
| AA/CC/TT/GA | 1 (0.3) | 1 (0.3) | 0.672 (0.039-11.464) | 0.784 | 0.909 |
| AT/TT/CC/GG | 7 (1.9) | 14 (3.6) | 1.415 (0.472-4.239) | 0.535 | 0.835 |
| AT/TT/CC/GA | 20 (5.3) | 19 (4.9) | 0.648 (0.269-1.560) | 0.333 | 0.753 |
| AT/TT/CC/AA | 4 (1.1) | 10 (2.6) | 1.739 (0.471-6.425) | 0.407 | 0.753 |
| AT/TT/CT/GG | 10 (2.7) | 12 (3.1) | 0.727 (0.249-2.122) | 0.560 | 0.835 |
| AT/TT/CT/GA | 19 (5.1) | 26 (6.7) | 0.964 (0.416-2.234) | 0.933 | 0.933 |
| AT/TT/CT/AA | 5 (1.3) | 6 (1.5) | 0.809 (0.215-3.054) | 0.755 | 0.909 |
| AT/TT/TT/GG | 5 (1.3) | 5 (1.3) | 0.545 (0.129-2.303) | 0.409 | 0.753 |
| AT/TT/TT/GA | 3 (0.8) | 4 (1.0) | 0.874 (0.174-4.392) | 0.870 | 0.909 |
| AT/TT/TT/AA | 1 (0.3) | 1 (0.3) | 0.750 (0.042-13.306) | 0.844 | 0.909 |
| AT/TC/CC/GG | 6 (1.6) | 4 (1.0) | 0.454 (0.113-1.829) | 0.267 | 0.753 |
| AT/TC/CC/GA | 5 (1.3) | 12 (3.1) | 1.760 (0.512-6.042) | 0.369 | 0.753 |
| AT/TC/CC/AA | 4 (1.1) | 3 (0.8) | 0.504 (0.101-2.518) | 0.404 | 0.753 |
| AT/TC/CT/GG | 12 (3.2) | 3 (0.8) | 0.170 (0.042-0.683) | 0.013 | 0.580 |
| AT/TC/CT/GA | 15 (4.0) | 7 (1.8) | 0.319 (0.109-0.938) | 0.038 | 0.581 |
| AT/TC/CT/AA | 2 (0.5) | 6 (1.5) | 2.053 (0.373-11.316) | 0.409 | 0.753 |
| AT/TC/TT/GG | 4 (1.1) | 2 (0.5) | 0.343 (0.056-2.095) | 0.246 | 0.753 |
| AT/TC/TT/GA | 2 (0.5) | 3 (0.8) | 1.141 (0.163-7.973) | 0.895 | 0.914 |
| AT/TC/TT/AA | 0 (0.0) | 1 (0.3) | N/A | N/A | N/A |
| AT/CC/CC/GG | 2 (0.5) | 0 (0.0) | N/A | N/A | N/A |
| AT/CC/CC/GA | 1 (0.3) | 2 (0.5) | 1.493 (0.118-18.942) | 0.757 | 0.909 |
| AT/CC/CC/AA | 0 (0.0) | 1 (0.3) | N/A | N/A | N/A |
| AT/CC/CT/GA | 1 (0.3) | 0 (0.0) | N/A | N/A | N/A |
| AT/CC/CT/AA | 1 (0.3) | 0 (0.0) | N/A | N/A | N/A |
| AT/CC/TT/AA | 0 (0.0) | 1 (0.3) | 2.741 (0.280-26.784) | 0.386 | 0.753 |
| TT/TT/CC/GG | 1 (0.3) | 4 (1.0) | 0.134 (0.015-1.244) | 0.077 | 0.709 |
| TT/TT/CC/GA | 5 (1.3) | 1 (0.3) | 1.448 (0.119-17.624) | 0.772 | 0.909 |
| TT/TT/CC/AA | 1 (0.3) | 2 (0.5) | N/A | N/A | N/A |
| TT/TT/CT/GG | 0 (0.0) | 3 (0.8) | N/A | N/A | N/A |
| TT/TT/CT/GA | 2 (0.5) | 4 (1.0) | 1.394 (0.230-8.435) | 0.718 | 0.909 |
| TT/TT/CT/AA | 0 (0.0) | 4 (1.0) | N/A | N/A | N/A |
| TT/TT/TT/GA | 1 (0.3) | 4 (1.0) | 2.647 (0.273-25.706) | 0.401 | 0.753 |
| TT/TT/TT/AA | 1 (0.3) | 1 (0.3) | 0.772 (0.042-14.087) | 0.861 | 0.909 |
| TT/TC/CC/GG | 0 (0.0) | 1 (0.3) | N/A | N/A | N/A |
| TT/TC/CC/GA | 1 (0.3) | 0 (0.0) | N/A | N/A | N/A |
| TT/TC/CT/GG | 1 (0.3) | 1 (0.3) | 0.733 (0.042-12.863) | 0.832 | 0.909 |
| TT/TC/CT/GA | 0 (0.0) | 2 (0.5) | N/A | N/A | N/A |
| TT/CC/CT/GA | 0 (0.0) | 2 (0.5) | N/A | N/A | N/A |
| *FGG* rs1049636T>C/*PECAM1* rs2812C>T/*PEAR1* rs12137505G>A | | | | | |
| TT/CC/GG | 27 (7.2) | 46 (11.8) | | | |
| TT/CC/GA | 67 (17.9) | 55 (14.1) | 0.485 (0.267-0.880) | 0.017 | 0.130 |
| TT/CC/AA | 22 (5.9) | 22 (5.7) | 0.589 (0.276-1.258) | 0.172 | 0.352 |
| TT/CT/GG | 32 (8.5) | 26 (6.7) | 0.466 (0.229-0.949) | 0.035 | 0.201 |
| TT/CT/GA | 45 (12.0) | 66 (17.0) | 0.884 (0.479-1.631) | 0.693 | 0.766 |
| TT/CT/AA | 17 (4.5) | 19 (4.9) | 0.657 (0.292-1.481) | 0.311 | 0.488 |

| | | | | | |
|---|---|---|---|---|---|
| TT/TT/GG | 11 (2.9) | 9 (2.3) | 0.428 (0.153-1.196) | 0.105 | 0.345 |
| TT/TT/GA | 8 (2.1) | 19 (4.9) | 1.354 (0.517-3.542) | 0.537 | 0.727 |
| TT/TT/AA | 5 (1.3) | 3 (0.8) | 0.356 (0.077-1.650) | 0.189 | 0.352 |
| TC/CC/GG | 13 (3.5) | 12 (3.1) | 0.547 (0.218-1.373) | 0.199 | 0.352 |
| TC/CC/GA | 24 (6.4) | 28 (7.2) | 0.691 (0.335-1.426) | 0.318 | 0.488 |
| TC/CC/AA | 8 (2.1) | 6 (1.5) | 0.437 (0.137-1.397) | 0.163 | 0.352 |
| TC/CT/GG | 24 (6.4) | 14 (3.6) | 0.341 (0.151-0.769) | 0.010 | 0.130 |
| TC/CT/GA | 32 (8.5) | 22 (5.7) | 0.404 (0.196-0.833) | 0.014 | 0.130 |
| TC/CT/AA | 9 (2.4) | 13 (3.3) | 0.844 (0.318-2.241) | 0.733 | 0.766 |
| TC/TT/GG | 6 (1.6) | 6 (1.5) | 0.588 (0.172-2.013) | 0.398 | 0.572 |
| TC/TT/GA | 7 (1.9) | 5 (1.3) | 0.440 (0.126-1.538) | 0.198 | 0.352 |
| TC/TT/AA | 4 (1.1) | 1 (0.3) | 0.146 (0.015-1.404) | 0.096 | 0.345 |
| CC/CC/GG | 4 (1.1) | 1 (0.3) | 0.144 (0.015-1.367) | 0.091 | 0.345 |
| CC/CC/GA | 2 (0.5) | 5 (1.3) | 1.456 (0.261-8.110) | 0.669 | 0.766 |
| CC/CC/AA | 1 (0.3) | 1 (0.3) | 0.585 (0.034-9.923) | 0.710 | 0.766 |
| CC/CT/GG | 0 (0.0) | 4 (1.0) | N/A | N/A | |
| CC/CT/GA | 2 (0.5) | 3 (0.8) | 0.883 (0.139-5.633) | 0.896 | 0.896 |
| CC/CT/AA | 3 (0.8) | 1 (0.3) | 0.196 (0.019-1.984) | 0.168 | 0.352 |
| CC/TT/GG | 1 (0.3) | 0 (0.0) | N/A | N/A | |
| CC/TT/GA | 1 (0.3) | 1 (0.3) | 0.593 (0.035-9.913) | 0.716 | 0.766 |
| CC/TT/AA | 0 (0.0) | 1 (0.3) | N/A | N/A | |
| *ITGB3* rs3809865A>T/*GP1BA* rs6065C>T | | | | | |
| AA/CC | 202 (53.9) | 174 (44.7) | 1.000 (reference) | | |
| AA/CT | 29 (7.7) | 41 (10.5) | 1.651 (0.978 - 2.785) | 0.061 | 0.146 |
| AA/TT | 2 (0.5) | 3 (0.8) | 1.241 (0.188 - 8.207) | 0.823 | 0.945 |
| TA/CC | 101 (26.9) | 119 (30.6) | 1.359 (0.972 - 1.902) | 0.073 | 0.146 |
| TA/CT | 26 (6.9) | 23 (5.9) | 1.022 (0.559 - 1.866) | 0.945 | 0.945 |
| TA/TT | 2 (0.5) | 0 (0.0) | N/A | N/A | |
| TT/CC | 9 (2.4) | 22 (5.7) | 3.107 (1.382 - 6.987) | 0.006 | 0.036 |
| TT/CT | 3 (0.8) | 7 (1.8) | 2.606 (0.650 - 10.447) | 0.176 | 0.264 |
| TT/TT | 1 (0.3) | 0 (0.0) | N/A | N/A | |
| *PEAR1* rs822442C>A/*PEAR1* rs12137505G>A | | | | | |
| CC/GG | 115 (30.7) | 110 (28.3) | 1.000 (reference) | | |
| CC/GA | 61 (16.3) | 52 (13.4) | 0.898 (0.570 - 1.413) | 0.641 | 0.871 |
| CC/AA | 9 (2.4) | 7 (1.8) | 0.817 (0.293 - 2.278) | 0.700 | 0.871 |
| CA/GG | 3 (0.8) | 7 (1.8) | 2.462 (0.621 - 9.764) | 0.200 | 0.700 |
| CA/GA | 126 (33.6) | 140 (36.0) | 1.170 (0.819 - 1.672) | 0.388 | 0.871 |
| CA/AA | 25 (6.7) | 25 (6.4) | 1.052 (0.570 - 1.944) | 0.871 | 0.871 |
| AA/GG | 0 (0.0) | 1 (0.3) | N/A | N/A | |
| AA/GA | 1 (0.3) | 12 (3.1) | 12.661 (1.618 - 99.085) | 0.016 | 0.112 |
| AA/AA | 35 (9.3) | 35 (9.0) | 1.054 (0.616 - 1.804) | 0.848 | 0.871 |

RPL, recurrent pregnancy loss; AOR, adjusted odds ratio; adjusted by age.
*P*-value was calculated using logistic regression.

Supplementary Table S5. Clinical variables in RPL patients stratified by platelet-related gene polymorphisms status by ANOVA and Kruskal-Wallis test.

| Genotypes | CD56 NK cell (%) | CD3(pan T) (%) | CD4(helper T) (%) | CD8(suppressor T) (%) | CD19(B-Cell) (%) |
|---|---|---|---|---|---|
| | Mean ± SD (n) | Mean ± SD (n) | Mean ± SD (n) | Mean ± SD (n) | Mean ± SD (n) |
| *ITGB3* rs2317676A>G | | | | | |
| AA | 17.5±7.2 (117) | 67.1±8.8 (65) | 36.0±7.3 (65) | 27.7±8.5 (65) | 12.4±5.1 (65) |
| AG | 17.5±9.5 (48) | 66.2±9.4 (25) | 36.7±8.1 (25) | 26.8±6.1 (25) | 13.0±4.2 (25) |
| GG | 15.2±5.1 (11) | 69.3±5.4 (6) | 38.8±5.7 (6) | 26.2±5.7 (6) | 14.0±5.2 (6) |
| *P* | 0.581* | 0.798* | 0.660 | 0.961* | 0.674 |
| *ITGB3* rs3809865A>T | | | | | |
| AA | 17.80 ± 8.67 (103) | 66.67 ± 10.33 (52) | 37.48 ± 8.10 (52) | 27.29 ± 8.19 (52) | 13.19 ± 5.17 (52) |
| AT | 16.35 ± 5.85 (57) | 67.24 ± 6.22 (34) | 34.66 ± 6.36 (34) | 28.45 ± 7.09 (34) | 12.74 ± 4.30 (34) |
| TT | 18.23 ± 7.91 (16) | 68.00 ± 7.54 (10) | 36.60 ± 6.40 (10) | 23.70 ± 7.27 (10) | 9.60 ± 4.50 (10) |
| *P* | 0.668 | 0.893 | 0.227* | 0.237* | 0.102* |
| *FGG* rs1049636T>C | | | | | |
| TT | 16.56 ± 7.29 (116) | 68.70 ± 6.29 (61) | 36.79 ± 7.17 (61) | 27.47 ± 6.81 (61) | 13.59 ± 4.70 (61) |
| TC | 18.97 ± 8.65 (50) | 63.62 ± 11.68 (32) | 35.99 ± 8.11 (32) | 26.61 ± 9.34 (32) | 11.04 ± 5.05 (32) |
| CC | 18.77 ± 8.24 (10) | 68.67 ± 7.23 (3) | 32.67 ± 4.04 (3) | 32.00 ± 8.89 (3) | 11.00 ± 1.00 (3) |
| *P* | 0.267 | 0.026 | 0.604* | 0.506* | **0.034** |
| *FGG* rs2066865T>C | | | | | |
| TT | 16.73 ± 7.91 (44) | 65.88 ± 8.36 (24) | 37.38 ± 7.44 (24) | 25.07 ± 4.74 (24) | 14.48 ± 5.49 (24) |
| TC | 17.21 ± 7.89 (87) | 68.10 ± 7.41 (52) | 36.32 ± 6.31 (52) | 28.01 ± 8.57 (52) | 12.11 ± 4.79 (52) |
| CC | 18.30 ± 7.57 (45) | 65.51 ± 11.93 (20) | 35.40 ± 9.91 (20) | 28.27 ± 8.29 (20) | 11.89 ± 3.92 (20) |
| *P* | 0.558 | 0.523 | 0.677* | 0.221 | 0.104* |
| *GP1BA* rs2243093T>C | | | | | |
| TT | 17.5±8.1 (96) | 65.9±10.1 (53) | 35.6±7.9 (53) | 26.5±7.1 (53) | 12.9±5.3 (53) |
| TC | 17.3±7.7 (69) | 68.2±6.8 (39) | 37.5±6.4 (39) | 27.9±8.5 (39) | 12.3±4.3 (39) |
| CC | 16.5±5.8 (11) | 69.5±0.0 (4) | 36.3±9.9 (4) | 31.8±8.7 (4) | 14.0±4.5 (4) |
| *P* | 0.974* | 0.403 | 0.447 | 0.372* | 0.725 |
| *GP1BA* rs6065C>T | | | | | |
| CC | 16.94 ± 7.73 (138) | 67.49 ± 8.54 (79) | 36.57 ± 7.66 (79) | 27.95 ± 7.82 (79) | 12.56 ± 4.61 (79) |
| CT | 18.94 ± 8.02 (37) | 66.11 ± 7.99 (16) | 36.28 ± 5.67 (16) | 24.73 ± 7.17 (16) | 13.59 ± 5.95 (16) |
| TT | 18.00 (1) | 43.00 (1) | 24.00 (1) | 20.00 (1) | 5.00 (1) |
| *P* | 0.319 | 0.151 | 0.243* | 0.205* | 0.217* |
| *PECAM1* rs2812C>T | | | | | |
| CC | 17.99 ± 8.67 (77) | 66.74 ± 8.14 (37) | 37.32 ± 7.25 (37) | 27.05 ± 8.20 (37) | 14.55 ± 5.30 (37) |
| CT | 16.94 ± 6.81 (84) | 67.12 ± 8.84 (48) | 35.13 ± 6.66 (48) | 28.12 ± 7.56 (48) | 11.46 ± 4.36 (48) |
| TT | 16.57 ± 8.47 (15) | 67.43 ± 10.88 (11) | 38.77 ± 10.33 (11) | 24.79 ± 7.24 (11) | 11.49 ± 3.81 (11) |
| *P* | 0.685 | 0.624 | 0.212* | 0.234 | 0.009* |
| *PEAR1* rs822442C>A | | | | | |
| CC | 17.75 ± 7.30 (65) | 69.24 ± 6.68 (42) | 38.44 ± 7.11 (42) | 27.20 ± 5.91 (42) | 13.19 ± 5.45 (42) |
| CA | 17.71 ± 8.15 (94) | 65.74 ± 7.71 (44) | 34.63 ± 6.48 (44) | 27.13 ± 7.97 (44) | 12.34 ± 4.33 (44) |
| AA | 14.02 ± 7.18 (17) | 63.20 ± 16.48 (10) | 35.59 ± 10.61 (10) | 28.71 ± 13.10 (10) | 11.80 ± 4.92 (10) |
| *P* | 0.130 | 0.131 | 0.053* | 0.808 | 0.610* |
| *PEAR1* rs12137505G>A | | | | | |
| GG | 19.33 ± 7.88 (49) | 67.88 ± 7.11 (33) | 38.69 ± 7.12 (33) | 25.83 ± 5.63 (33) | 13.18 ± 5.81 (33) |
| GA | 17.25 ± 7.63 (101) | 65.89 ± 9.81 (55) | 35.01 ± 7.57 (55) | 27.86 ± 8.93 (55) | 12.30 ± 4.38 (55) |
| AA | 14.14 ± 7.37 (26) | 71.11 ± 5.44 (8) | 36.41 ± 5.57 (8) | 29.79 ± 6.31 (8) | 12.93 ± 4.31 (8) |
| *P* | **0.008** | 0.372 | 0.078* | 0.354 | 0.709* |

ANOVA, analysis of variance, SD, standard deviation.
Calculated using the Kruskal-Wallis test. *Calculated using ANOVA.

Supplementary Table S6. Information for genotyping of nine platelet activation and coagulation related gene polymorphisms.

| Gene | rs number | Region | Method | Primer sequence | Product size | Annealing temperature | Restriction enzyme |
|---|---|---|---|---|---|---|---|
| *ITGB3* | rs2317676 | 3' UTR | Taq-man | Forward 5'- GTT CTC TCG CAA GGG AAG T -3'<br>Reverse 5'- TGT CTC TCA TGA CCA AAT GCT -3' | 284bp | 52 °C | |
| | rs3809865 | 3' UTR | RFLP | Forward 5'- TCC TAA TTC CAC ACC CTC ACT GC -3'<br>Reverse 5'- GTA CCC TGC CCA AGT CTG TG -3' | 520bp | 56 °C | *Kpn*I |
| *FGG* | rs2066865 | 500bp downstream | RFLP | Forward 5'- CCT ATT GGA CAA TGG ACT TGC -3'<br>Reverse 5'- GTT GTG TGG CTG TGA CTC -3' | 399bp | 54 °C | *Dde*I |
| | rs1049636 | 3' UTR | Taq-man | Forward 5'- CGG TGG TAT TCC ATG AAG A -3'<br>Reverse 5'- CCA TTG AAG GCT AAA TGT CC -3' | 290bp | 49 °C | |
| *GP1BA* | rs2243093 | 5’ UTR | RFLP | Forward 5'- GTT CTC TCG CAA GGG AAG T -3'<br>Reverse 5'- TGT CTC TCA TGA CCA AAT GCT -3 | 407bp | 54 °C | *Ava*II |
| | rs6065 | missense | Taq-man | Forward 5'- AAT CAG CTG CAA AGC CTG -3'<br>Reverse 5'- GAT CTC ACA GTT GCA TAA CCA G -3' | 360bp | 51 °C | |
| *PECAM1* | rs2812 | 3' UTR | Taq-man | Forward 5'- TGT GGA GGG AAG CGC ACA G -3'<br>Reverse 5'- GCA GGG TGG TGT GGT TAT CAT -3' | 165bp | 55 °C | |
| *PEAR1* | rs822442 | missense | Taq-man | Forward 5'- GGG CCC CAT TTC TAG AGG AAG - 3'<br>Reverse 5'- AGG AAA GAA GTC AGG CCG CAT - 3' | 226bp | 55 °C | |
| | rs12137505 | missense | Taq-man | Forward 5'- GAA GGA GGG TAT GTG GGA AAT TC - 3'<br>Reverse 5'- TTG CAT TTC TCT CCA CCA GG - 3' | 334bp | 55 °C | |
| *PROC* | rs1799809 | upstream | Taq-man | Forward 5'- TTG TGC TGA TCT TGG GCA AAC -3'<br>Reverse 5'- GCC TGG AGT TCG AGT TAA TCC -3' | 309bp | 54 °C | |
| *PROCR* | rs9574 | 3' UTR | Taq-man | Forward 5'- ACT CCT TGG GGG CCT ATT CT -3'<br>Reverse 5'- TCT GGC TTC ACA GTG AGC TG -3' | 333bp | 54 °C | |

UTR, untranslated region; RFLP, restriction fragment length polymorphism.

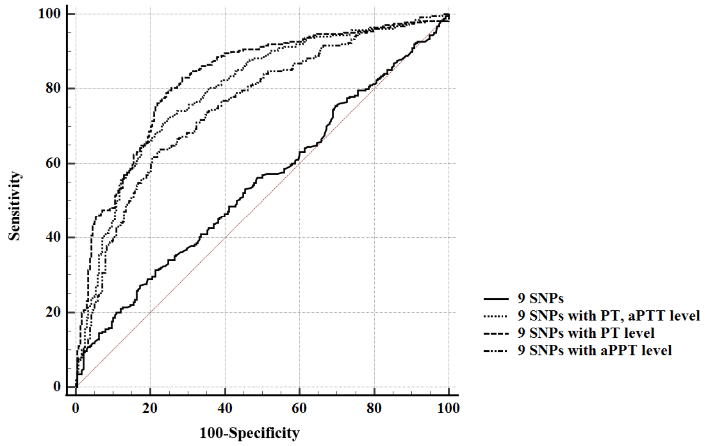


**Supplementary Figure S1. Receiver-operating characteristics (ROC) curve for the genetic and clinical combined model in RPL risk.** genetic risk score (9 SNPs: *ITGB3* rs2317676 A>G, rs3809865 A>T; *FGG* rs1049636 T>C, rs2066865 T>C; *GP1BA* rs2243093 T>C, rs6065 C>T; *PECAM1* rs2812 C>T; *PEAR1* rs822442 C>A, rs12137505 G>A), AUC 0.546 (0.502–0.589); combined risk score (9 SNPs and PT levels), AUC 0.826 (0.791–0.858); combined risk score (9 SNPs and aPTT levels), AUC 0.752 (0.713–0.788); combined risk score (9 SNPs, PT and aPTT levels), AUC 0.794 (0.757-0.828). The AUC is significantly larger for the combined risk score than for the genetic risk score; *P*-value < 0.001.
